# Liquid Metal-Based Epidermal Flexible Sensor for Wireless Breath Monitoring and Diagnosis Enabled by Highly Sensitive SnS_2_ Nanosheets

**DOI:** 10.34133/2021/9847285

**Published:** 2021-06-17

**Authors:** Yifan Huang, Fan Yang, Sanhu Liu, Rongguo Wang, Jinhong Guo, Xing Ma

**Affiliations:** ^1^ National Key Laboratory of Science and Technology on Advanced Composites in Special Environments, Harbin Institute of Technology, Harbin 150086, China; ^2^ Sauvage Laboratory for Smart Materials, School of Materials Science and Engineering, Harbin Institute of Technology (Shenzhen), Shenzhen 518055, China; ^3^ Shenzhen STRONG Advanced Materials Research Institute Co., Ltd., China; ^4^ School of Communication and Information Engineering, University of Electronic Science and Technology of China, Chengdu 611731, China; ^5^ Shenzhen Key Laboratory of Flexible Printed Electronics Technology, Harbin Institute of Technology (Shenzhen), Shenzhen 518055, China; ^6^ Shenzhen Bay Laboratory, No. 9 Duxue Road, Shenzhen 518055China

## Abstract

Real-time wireless respiratory monitoring and biomarker analysis provide an attractive vision for noninvasive telemedicine such as the timely prevention of respiratory arrest or for early diagnoses of chronic diseases. Lightweight, wearable respiratory sensors are in high demand as they meet the requirement of portability in digital healthcare management. Meanwhile, high-performance sensing material plays a crucial role for the precise sensing of specific markers in exhaled air, which represents a complex and rather humid environment. Here, we present a liquid metal-based flexible electrode coupled with SnS_2_ nanomaterials as a wearable gas-sensing device, with added Bluetooth capabilities for remote respiratory monitoring and diagnoses. The flexible epidermal device exhibits superior skin compatibility and high responsiveness (1092%/ppm), ultralow detection limits (1.32 ppb), and a good selectivity of NO gas at ppb-level concentrations. Taking advantage of the fast recovery kinetics of SnS_2_ responding to H_2_O molecules, it is possible to accurately distinguish between different respiratory patterns based on the amount of water vapor in the exhaled air. Furthermore, based on the different redox types of H_2_O and NO molecules, the electric signal is reversed once the exhaled NO concentration exceeds a certain threshold that may indicate the onset of conditions like asthma, thus providing an early warning system for potential lung diseases. Finally, by integrating the wearable device into a wireless cloud-based multichannel interface, we provide a proof-of-concept that our device could be used for the simultaneous remote monitoring of several patients with respiratory diseases, a crucial field in future digital healthcare management.

## 1. Introduction

Uninterrupted respiratory monitoring is critical in a clinical setting to improve the survival rate of patients with potential respiratory diseases. For instance, the widespread pandemic caused by the SARS-CoV-2 virus (COVID-19) has emerged as a major cause of respiratory failure [1–3]. Respiratory arrest is one of the main symptoms of epilepsy (SUDEP), brain injury, congestive heart, and failure and accounts for a high mortality [4–6]. Considering the paroxysm, real-time breath monitoring can greatly improve the survival of these diseases. Besides, real-time breath composition analysis is an effective method for chronic disease detection. For example, asthma is a chronic disease, and the symptoms of which are similar to respiratory tract infection or inflammation. The early treatment of asthma is often not timely or even misdiagnosed without proper early diagnosis or disease warning. The breath exhaled by humans is a complex mixture of more than 3000 compounds, and the exhaled NO is regarded as the main biomarker of asthma [7]. The early warning of these gas biomarkers is of particular importance for an early diagnosis of chronic diseases and can even be used to establish a personalized therapy schedule and/or guide daily healthcare management.

At present, respiratory monitoring sensors mainly rely on detecting changes in the physical signal of the exhaled gas flow, e.g., humidity, temperature, or pressure in the nose and mouth. For example, WS_2_ film, which has an electrochemical affinity for humidity, has been integrated with graphene electrodes and polydimethylsiloxane (PDMS) substrate to form an electronic skin (e-skin) that is capable of detecting respiration rates [8]. Furthermore, skin-like hybrid integrated circuits have been built to capture temperature changes in the inhaled and exhaled air [9]. A different study developed a pressure sensor consisting of composite films based on polyaniline hollow nanospheres integrated into face masks where they could be used for respiration monitoring [10]. Nanogenerators, such as pyroelectric nanogenerators (PyNGs), alveolus-inspired membrane sensors (AIMSs), or nanofiber-based triboelectric sensors (SNTSs), have been developed to serve as self-powered breath analyzers [11–15]. While these sensors typically focus on monitoring the frequency and amplitude of respiration, they cannot simultaneously analyze the exhaled air for biomarkers. The detection of gas biomarkers depends on specific techniques or instrumentation such as electrochemistry, surface-enhanced Raman scattering (SERS), chemiluminescence, colorimetric sensor, and infrared sensor [16–19]. For example, Il-Doo Kim group fabricated colorimetric dye-loaded nanofiber yarn which is sensitive to ppm-level H_2_S and NH_3_ biomarkers [15]. Zhou et al. utilized a mid-infrared hollow waveguide gas sensor to realize real-time measuring of CO_2_ isotopes [20]. Chen et al. developed a breath analysis approach based on SERS sensor to detect fourteen volatile organic compound (VOC) biomarkers [16]. In this context, the aforementioned physical factors of humidity, temperature, and pressure changes in the exhaled gas are regarded as mere interference factors which must be controlled or even eliminated to ensure an accurate analysis of the exhaled biomarkers. A device capable of real-time respiratory monitoring and simultaneous biomarker analysis would be highly desirable although its implementation faces some considerable challenges.

Real-time respiratory monitoring requires suitable gas sensing devices that should be lightweight and thus wearable to minimize any adverse effect on daily life activities [21, 22]. While the biomarker sensing material should be immune to interference from other gases and high levels of humidity (typically, exhaled air has >80% relative humidity), it should be highly sensitive to the target biomarker gas [23]. In this article, we report the fabrication of a wireless healthcare device based on a liquid metal (LM) electrode that is integrated with SnS_2_ gas sensing material to facilitate the uninterrupted remote monitoring of respiration while simultaneously providing the highly sensitive detection of certain breath biomarkers. An eutectic GaIn alloy-based LM with a low melting point delivers liquidus fluidity and metallic conductivity at room temperature, allowing for more flexible and reconfigurable electronics [16]. It is recognized as low toxicity, biosafety material both *in vitro* and *in vivo*, and its environmental friendliness is reported with high recycle efficiency [24–27]. By integrating the LM-based conductive pattern with ultrathin PET film, we fabricate an epidermal device that can be easily attached to the philtrum. Here, “epidermal” means that the sensor can be attached to the skin, but not detecting the gas emitted by the skin. The obtained device is skin compatible and compressible, while maintaining excellent conductivity. For the first time, rather than using metal oxide, we employed SnS_2_ two-dimensional (2D) nanosheets as NO sensing material, with NO being a noninvasive biomarker for many diseases such as anaphylactic purpura (AP), asthma, and myocarditis [28, 29]. Our device exhibits a superior response (197% at 200 ppb) and ultralow detection limit (1.32 ppb) for NO gas at room temperature. Moreover, the sensor exhibits an impressive selectivity against various exhaled biomarkers such as NH_3_, CH_4_, H_2_, ethanol, and acetone, while showing a remarkable resistance to interference by other gases. First-principle calculations based on density functional theory (DFT) suggest that the outstanding sensing performance can be attributed to the high adsorption energy, charge transfer, and the variation of the 2D SnS_2_ lamellar molecular structure. In addition, SnS_2_ nanomaterial interacts with the water vapor in the exhaled gas, which in turn allows for more accurate identifications of the respiratory state. Due to the different redox types of the H_2_O and NO molecules, the resistance stops from decreasing and starts to increase once the NO concentration exceeds around 58 ppb. This feature provides the device with the additional capacity to provide an early warning for lung disease. By equipping our device with Bluetooth functionality and cloud-based signal analysis, we provide a proof-of-concept for the multichannel real-time telemonitoring of the respiratory state, which holds great potential both in clinical applications and for remote healthcare management.

## 2. Results and Discussion

### 2.1. Fabrication of Epidermal LM Electrodes

**Figure 1:**
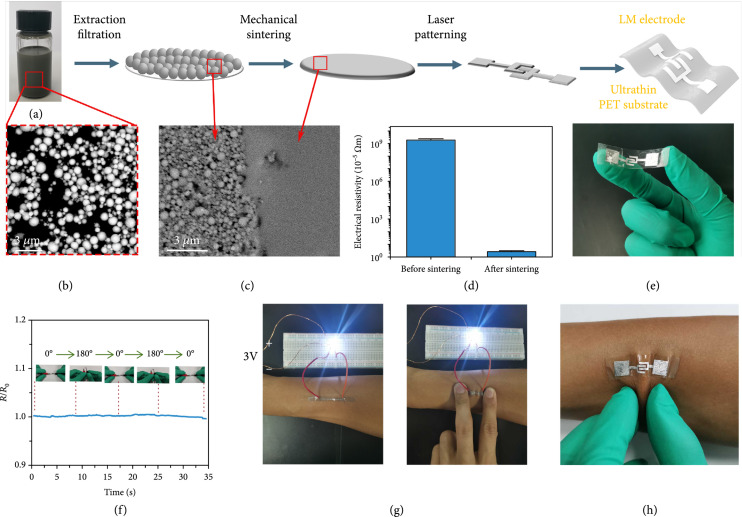
Fabrication of an LM-based epidermal electrode and characterization of its electrical properties. (a) Schematic of the fabrication process. (b) PVP stabilized LM nanoparticles suspended in ethanol. (c) SEM image of LM before and after mechanical sintering. (d) Electrical conductivity before and after mechanical sintering. (e) Photograph of the ultrathin electrode. (f) Change in conductivity during two consecutive bending processes from 0° to 180°. (g) If compressed by human skin, the LM wire maintains good conductivity and skin compatibility. (h) Flexibility and adaptability of the LM electrode to the human skin.

The flexible epidermal electrode is fabricated by integrating an LM nanoink-based conductive pattern onto a flexible PET film (Figure 1(a)). Firstly, the eutectic gallium-indium (EGaIn) alloy (melting point at 15°C) is dispersed into nanoparticles (average size 636.9±6.4 nm) (Figure 1(b) and Figure S1) by probe sonication assisted by polyvinyl pyrrolidone (PVP) as surfactant. After filtrating the EGaIn nanoparticles onto filter paper where they form a uniform film, they are mechanically sintered to restore their electric conductivity [30]. This destroys the oxide shell of the LM nanoparticle, and the conductive LM cores are fused together forming a flat film (Figure 1(c)). The sintering reduced the resistivity by nine orders of magnitude to $2.74\times 10^{-5}$ *Ω*m, a value that is comparable to common alloys and carbon materials (Figure 1(d)) [31–33]. The conductive film is finally laser patterned into electrodes as required and transferred onto transparent ultrathin polyethylene terephthalate (PET) film (0.02 mm) to yield a flexible LM-based electrode for integration into the gas sensor (Figure 1(e)).

To examine how the conductivity of the LM-based electrode varies when exposed to simulated human movement, we bent the electrode from 0° to 180° which resulted in only small changes in electrical resistance of less than 0.5% (Figure 1(f)). We then attached the wire onto the skin of an arm. When attached to human skin, the electrode maintained good conductivity even when bent of compressed (Figure 1(g)). We further measured the resistance change of the liquid metal electrode accurately. The relative change of resistance is only 0.17% (Figure S2). The stable conductivity gives the credit to the high fluidity of liquid metal, which avoid cracks when the circuit deforms. Besides, there is no obvious change for the conductivity of the LM electrode in 15 days (Figure S3), owing to the protection of the dense gallium oxide “skin” on the surface. To further explore its utility in wearable applications for respiratory gas sensing, the as-prepared epidermal electrode was attached to human skin where it showed good resilience to physical straining and maintained good adhesion (Figure 1(h)) which is promising for possible future applications as a wearable gas sensor.

### 2.2. Synthesis of SnS_2_ and Gas Sensing Performance

**Figure 2:**
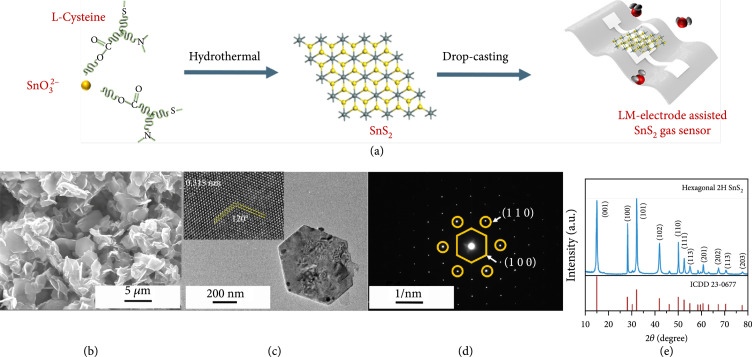
Synthesis and characterization of SnS_2_ 2D nanomaterial in gas sensing applications. (a) Schematic illustration of the hydrothermal synthesis of SnS_2_ and subsequent fabrication of the gas sensor using the drop-casting method. (b) SEM image of stacked SnS_2_ 2D nanolayers. (c) TEM image and HRTEM image (inset) of the hexagonal structure of an SnS_2_ single nanolayer. (d) Fast Fourier transform (FFT) pattern of the SnS_2_. (e) X-ray diffraction (XRD) pattern of the SnS_2_ confirming its 2H structure.

SnS_2_ is a relatively abundant and environmentally friendly semiconductor material with a wide bandgap of 2.1 eV [33–35]. It has been reported to be a highly sensitive gas sensing material capable of detecting NO_2_ and NH_3_ at ppb-level concentrations [36–38]. Here, for the first time, we explore the capabilities of SnS_2_ for sensing NO molecules. The SnS_2_ gas sensing material was synthesized using a slightly modified version of a previously reported hydrothermal method (Figure 2(a)) [39]. SnO_3_^2-^ and L-cysteine were chosen as source materials for Sn and S, respectively. The microstructure of the obtained SnS_2_ nanomaterials was drop-cast onto a substrate (Figure 2(b)) resulting in randomly overlapping hexagonal layers (Figure 2(c)). We measured the lattice fringe spacing as 0.315 nm on both the (100) and (010) lattice planes of hexagonal SnS_2_. Interaxial angles were consistently 120° (Figure 2(c), inset) [40]. The fast Fourier transform pattern of this region can be indexed to 2H-SnS_2_ along the (100) and (110) zone axes (Figure 2(d)) [41]. The crystal phase was identified using X-ray diffraction (XRD) (Figure 2(e)). The observed diffraction peaks are in agreement with the hexagonal 2H SnS_2_ structure (ICDD 23-0677). This structure belongs to the P3m1 space group where three atoms extend over only one monolayer in a unit cell [37]. These characterizations prove the successful fabrication of the SnS_2_ 2D nanomaterials.

**Figure 3:**
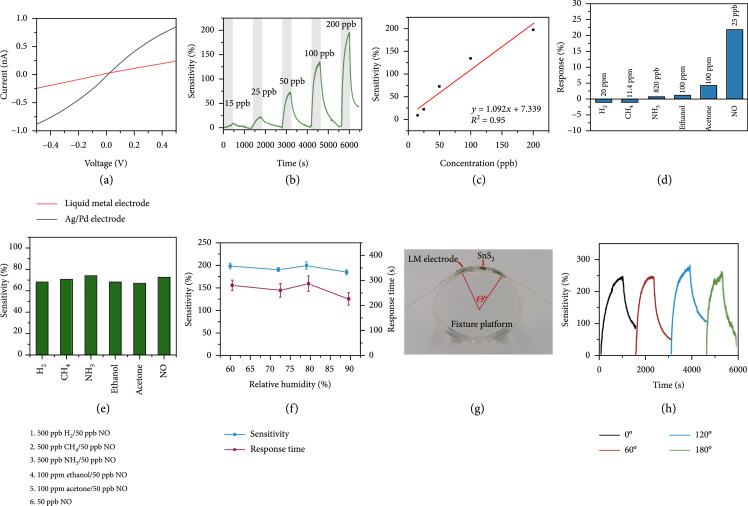
NO gas sensing performance of the LM@SnS_2_-based sensor. (a) Comparing the voltammetric curves of our SnS_2_@LM electrode and a commercially available Ag/Pd electrode. (b) Dynamic response to NO gas concentrations ranging from 15 ppb to 200 ppb at room temperature. (c) Linear regression of sensitivity versus concentration data. (d) Measured cross-talk of the sensor to NO and other exhaled interfering gases. (e) Sensing performance of the device when simultaneously exposed to 50 ppb NO and another exhaled interfering gases at various concentrations. (f) Sensing behavior with 200 ppb NO gas for relative humidities ranging from 60% to 90%. (g) The platform used for flexibility testing. (h) The dynamic response to 250 ppb NO gas at different angles of bending.

The 2D nanomaterials of the as-prepared SnS_2_ were drop-cast onto the LM electrodes. Figure 3(a) illustrates the I–V curves of the integrated gas sensing device. Due to the higher work function, a commercially available Ag/Pd electrode-based device displayed rectifying characteristics, forming Schottky junctions between metallic electrodes and the sensing material [35]. This potential barrier could hinder the flow of charge carriers and thereby affect sensing performance [42]. In contrast, our epidermal LM-based device exhibited linear characteristics, indicating ohmic contact between the sensing material and the electrode. The band energy diagram is shown in Figure S4. The work function of SnS_2_ and EGIA is reported to be 5.1 eV and 4.1-4.2 eV, respectively [40, 43]. Since the work function of EGIA is higher than that of SnS_2_, ohmic contact is forming. As a result, our LM electrode would not affect the sensing behavior of SnS_2_.

To examine the ability of the LM electrode to sense NO, we fabricated a gas-testing platform using a homemade chamber (Scheme S1). To mimic the actual human respiratory environment, we chose air as background vapor and performed all experiments at room temperature. The target gas concentration was controlled by injecting certain volumes of NO gas into the chamber using high-precision syringes. The sensors ability to detect any change in NO concentration was estimated by measuring changes in the resistance of the sensing material prior ($R_{0}$) and after ($R_{g}$) having injected the target gas. We defined the sensitivity as $\left(R_{g}-R_{0}\right)/R_{0}$ and the response time as the time required to reach 90% of the full sensitivity. As an n-type semiconductor, SnS_2_ could potentially adsorb NO gas and electrons could then transfer from SnS_2_ layers to NO molecules (forming NO^-^) based on the charge transfer mechanism [44]. Thus the charge concentration of SnS_2_ would decrease while the resistance dramatically increases. Figure 3(b) shows the dynamic performance of the device responding to ppb-level concentrations of NO gas that increased from 15 ppb to 200 ppb, thus covering the typical range of human-exhaled NO concentrations. The device exhibited a 197% sensitivity to 200 ppb with a response time of 223 s and easily recovered its initial resistance once we had stopped injecting NO. In contrast to most other NO sensors, our LM-based electrode performed rather well at low concentrations and could detect NO levels as low as 15 ppb with a sensitivity of 8.7% [45–54]. The sensitivity of our sensor exhibits a strong linear correlation with the gas concentration (Figure 3(c), $R^{2}=0.95$). The limit of detection (LOD) is usually defined as the target gas concentration at which the sensor is still capable to produce a signal that is three time higher than the sensor’s noise level. The LOD of our device was determined as 1.32 ppb. When comparing the sensitivity, experimental LOD, and operating temperature of our epidermal device to other NO sensors that had been reported in the literature (Table S1), we found that our sensor outperforms other NO sensors in terms of sensitivity, i.e., detecting lower ppb-level concentrations with higher sensitivity, a lower detection limit, and—more importantly—its good performance at room temperature which is a crucial requirement for NO gas detection in most respiratory applications.

Selectivity is crucial for avoiding false alarms that can be caused by other interfering gases, especially in the monitoring of human exhaled breath which is composed of a variety of gas mixtures. Five other commonly exhaled gas molecules are selected to test the selectivity of the device: H_2_ (biomarker of pancreatic diseases), CH_4_ (biomarker of gut diseases), NH_3_ (biomarker of oral, renal, and liver diseases), and volatile organic compounds (VOCs) such as ethanol (main component of wine which can be exhaled after drinking) and acetone (biomarker of diabetes mellitus) [7]. The concentration of H_2_, CH_4_, NH_3_, and NO is controlled at their clinically relevant concentrations: 20 ppm, 11.4 ppm, 820 ppb, and 25 ppb, respectively [7, 55, 56]. And the concentration of VOC is 100 ppm (much higher than exhaled concentration of healthy people) [57]. Compared with the response to NO, each of these tested interfering gases yielded a negligible sensing response (Figure 3(d)). In order to better simulate the situation of multiple exhaled gases detection, another experiment was carried out by injecting one of these five interfering gases along with NO, but at higher concentrations. While NO was maintained at 50 ppb, each of H_2_, CH_4_, and NH_3_ was injected to yield 500 ppb, while VOC was injected to yield 100 ppm. Thus, although the interfering gas concentration was between one and ten orders of magnitude higher than that of NO, the response to NO remained relatively constant at around 70% with only minor variations (Figure 3(e)). This indicates that our sensor is capable to accurately identify and measure the concentration of NO gas and is relatively immune to the presence of other interfering gas biomarkers.

Water vapor (H_2_O) is one of the main components of human exhalation, and the relative humidity in exhaled gas is around 85% [7], i.e., the concentration of water vapor is much higher than the concentration of common gas biomarkers (generally of the order of ppm). Unlike N_2_ which is present at high concentrations in exhaled breath and that possess a stable electronic structure, H_2_O molecules easily become ionized to H_2_O^+^. If the electrons from the ionization of water vapor are passed on to SnS_2_, the resistance of SnS_2_ would change. In order to assess the impact of this process on the resistance of our electrode, we exposed our prototypes to high relative humidities of 70% to 90% (Figure S5). The resistance of electron-doped SnS_2_ was indeed reduced, showing an opposite electronic signal response compared to NO. Since the sensor response is correlated to H_2_O concentration, water vapor can act as a marker of respiration state. We also tested whether the sensor response to NO differed for different relative humidities (Figure 3(f)). We found that even when the relative humidity was increased to 90%, there was only a minor decrease in sensor sensitivity (<10%) for NO concentrations of 200 ppb. Higher relative humidities also decreased the response time by 19.2%, which may be due to the fact that the ionized form of SnS_2_ is more abundant at higher humidities and can therefore provide its additional electron to the adsorbed NO molecules. Thus, while the detection of NO by our gas sensor would not be affected by the amount of exhaled water vapor, the amount of vapor can be used to identify the respiratory state, which endows the devices with an additional functionality in respiratory monitoring.

O_2_ and CO_2_ are the two main gases which are inhaled or exhaled by the human body, respectively. As shown in Figures S6 and S7, we investigate the influence of the concentration change of the two gases on the sensing response. The response to 5% concentration of O_2_ is lower than 7.5% (Figure S6), much lower than the response to 25 ppb of NO (21.6%). Therefore, the change of oxygen concentration has little effect on the sensing performance. The response to 4% concentration of CO_2_ is about 62% (Figure S7). Since both NO and CO_2_ are oxidizing gases, the contribution from CO_2_ would decrease the threshold for the alarm function of NO sensing (details are discussed in cloud-based remote breath monitoring and diagnosis section). We also calculated the adsorption parameters of SnS_2_ layer to CO molecule to evaluate the sensing response to CO biomarker. The adsorption energy and charge transfer are far less than that to NO and H_2_O; thus, we can infer that the sensor is not sensitive to CO (Figures S8 and S9).

Considering the temperature difference of ambient environment and body temperature, we further tested the temperature effect on the sensing signal (Figure S10). The relative resistance variation of the sensor is only 1.997% when changing temperature from 24.5°C to 34.3°C, indicating that the temperature change caused by respiration has negligible effect on the performance of the sensor. Besides, like all wearable devices, epidermal sensors often need to operate under conditions of physical strain (e.g., being bent when adapting to the deformation of the skin as the person moves). As mechanical deformations can potentially increase the contact area between SnS_2_ nanosheets and NO molecules, the sensor sensitivity can increase by up to 10% when being bent (Figure 3(h)). Furthermore, while mechanical strain can decrease the amount of overlap between the different SnS_2_ layers and thereby increase the sensor noise (manifested as increasing jitter in Figure 3(h)) as the bending angle is increased, the sensor still maintains a high signal-to-noise ratio for all tested angles.

Considering the photosensitivity of the SnS_2_ nanomaterial, we investigated the capacity of the epidermal sensor for photoelectric sensing [58, 59]. The band gas of the SnS_2_ layer was confirmed to be 2.4 eV by UV-vis spectra (Figure S11). Detailed results and a discussion can be found in the supplementary information (Figures S5-S8). In short, our device produced a significant photocurrent ($I_{p}$) in response to blue (450 nm), green (532 nm), and red (650 nm) light with blue light triggering the largest response (Figure S12). The photocurrent was highly correlated to the power density, P, as $I_{p}=143.55\times P^{0.51}$ ($R^{2}=0.9906$) (Figures S13 and S14). Previous studies have found that photocurrents can affect the gas sensing performance [40, 60]. We therefore examined the effect of photo-generated currents on the gas sensing behavior of our sensor to 200 ppb of NO gas under different illumination conditions. Red light with an intensity of 3 mW/cm^2^ boosted the sensitivity by a factor 2.14 compared to dark conditions (Figure S15). We expect that future designs of the sensor could include a remote light control mechanism to achieve on-demand regulation of the sensor’s performance.

### 2.3. Theoretical Models of Gas Sensing Behavior

To elucidate the underlying mechanisms behind the good gas sensing performance of our LM-SnS_2_ sensor, we performed some numerical simulations of the molecule-surface binding energy, charge transfer, and adsorption distance employing density functional theory (DFT). The adsorption energy was calculated from $\text{Eads}\left[\text{Sn}{\text{S}}_{2}+\text{gas}\right]=E\left[\text{compound}\right]-E\left[\text{Sn}{\text{S}}_{2}\right]-E\left[\text{gas}\right]$, where $E\left[\text{compound}\right]$ is the total energy of the sensor supercell and the target gas molecule after adsorption, $E\left[\text{Sn}{\text{S}}_{2}\right]$ is the total energy of the SnS_2_ layer, and $E\left[\text{gas}\right]$ is the energy of the adsorbed target gas molecule.

**Figure 4:**
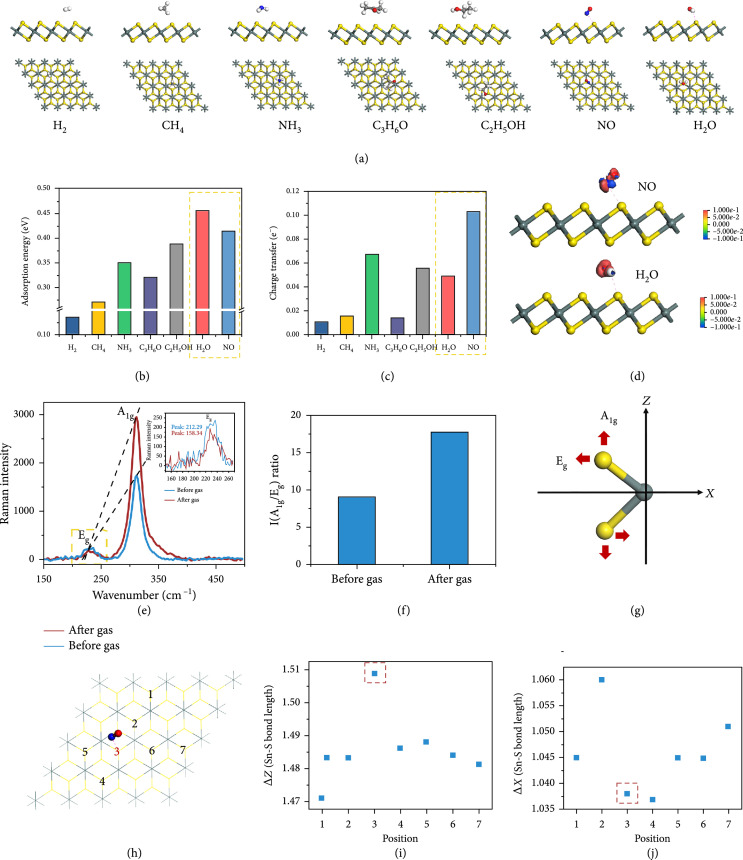
Theoretical interpretations based on first-principle calculations. (a) Optimized target gas molecules are adsorbed onto the SnS_2_4×4×1 supercell. Calculated (b) molecular surface adsorption energies and (c) charge transfer. (d) Charge density difference between NO and H_2_O molecules after being adsorbed onto SnS_2_, where the red and blue regions represent electron loss and gain, respectively. (e) Raman spectrum of SnS_2_ before and after adsorbing NO gas and (f) the corresponding intensity ratio of A_1g_ and E_g_ modes. (g) Calculated deformation along the X- and Z-directions of the A_1g_ and E_g_ modes. (h) The seven chosen S atoms (S atom no. 3 is the NO adsorption site) and the positional change of the seven S atoms in the (i) Z- and (j) X-directions after NO adsorption.

Figure 4(a) shows the model of SnS_2_ adsorbing NO and the aforementioned interfering gases. The distance between the target gas molecules and the SnS_2_ surface ranges from 2.14 to 3.01 Å which is within the typical range for physisorption (Figure S16) [37]. The sensitivity of this process depends on the total charge transfer from the target gas to SnS_2_ that leads to the change in resistance. A key factor is the binding strength that determines the number of adsorbed molecules. The two highest binding energies were calculated for NO and H_2_O, which demonstrates their strong binding strength to the SnS_2_ surface relative to the other interfering gases (Figure 4(b)). The charge transfer between individual molecules is another key factor to explain the high sensitivity of the sensor. A Mulliken population analysis demonstrates how NO has a much higher charge density (0.103 e^-^) compared to the interfering gases (Figure 4(c)). This may be due to a more favorable Fermi energy of the SnS_2_ layers and the only partially occupied molecular orbitals of NO [37]. Furthermore, for respiratory monitoring applications, the sensors need to be able to complete the response and recovery during a single breathing cycle, i.e., the adsorption speed of the target gas at room temperature is a critical parameter. Our sensor was capable of much faster recovery kinetics for NO and H_2_O compared to reported values for other physisorption-based sensors (e.g., graphene, MoS2) [61, 62]. An adsorption distance greater than 2 Å facilitates desorption during thermal vibrations or gas flow disturbance. This effect together with the strong physical affinity of NO gas to SnS_2_ ensures the fast reaction and recovery kinetics of SnS_2_ layers for NO sensing even at room temperature.

Although the charge transfer for individual H_2_O molecule is low, the fact that the concentration of H_2_O in human breath is several orders of magnitude higher than the concentration of other biomarker gases means that H_2_O vapor can significantly affect the resistance of SnS_2_. Figure 4(d) shows the difference in charge density between NO and H_2_O adsorption on SnS_2_. As the charge transfer occurs in opposite directions, this produces opposite electrical signals in SnS_2_ (Figure 3(b)). These differing responses of SnS_2_ to the presence of H_2_O and NO enable the sensor to perform respiratory monitoring and disease warning simultaneously. That is, the resistance of SnS_2_ is reduced when in contact with water vapor and increased with increasing NO concentration.

The strong affinity of SnS_2_ for NO gas is also shown by the distortion of the molecular structure of SnS_2_ after gas adsorption. NO adsorption induced a deformation of the sandwiched structure of SnS_2_ layers. The Raman peaks have shifted after exposure to NO gas (Figure 4(e)). The A_1g_ (located at 312 cm^-1^) and E_g_ peaks (located at 232 cm^-1^) correspond to the vertical and horizontal plane vibrational modes of the Sn-S bonds, respectively [37]. As there was no other peak present after gas adsorption, this confirms the physisorption process. NO adsorption led to a significant increase in the intensity of the A_1g_ mode and a slight decrease in the E_g_ mode, which in turn led to an increase in the intensity ratio $I\left({\text{A}}_{1\text{g}}/{\text{E}}_{\text{g}}\right)$ from 9.04 to 17.72 (Figure 4(f)). This behavior corresponds to the SnS_2_ layers elongating in the vertical and compressing in the horizontal direction, with the vertical elongation being greater than the horizontal compression. This behavior is confirmed by our calculations regarding the deformations along the X- and Z-axes (Figure 4(g)). We also calculated the changes in position of sulfur atoms at seven different sites, with NO adsorption occurring at the site labeled 3 (Figure 4(h)). As a result of NO adsorption, this third sulfur atom exhibited the greatest displacement in both X- and Z-directions, proving the structural change of the SnS_2_ layers (Figures 4(i) and 4(j)).

### 2.4. Cloud-Based Remote Breath Monitoring and Diagnosis

**Figure 5:**
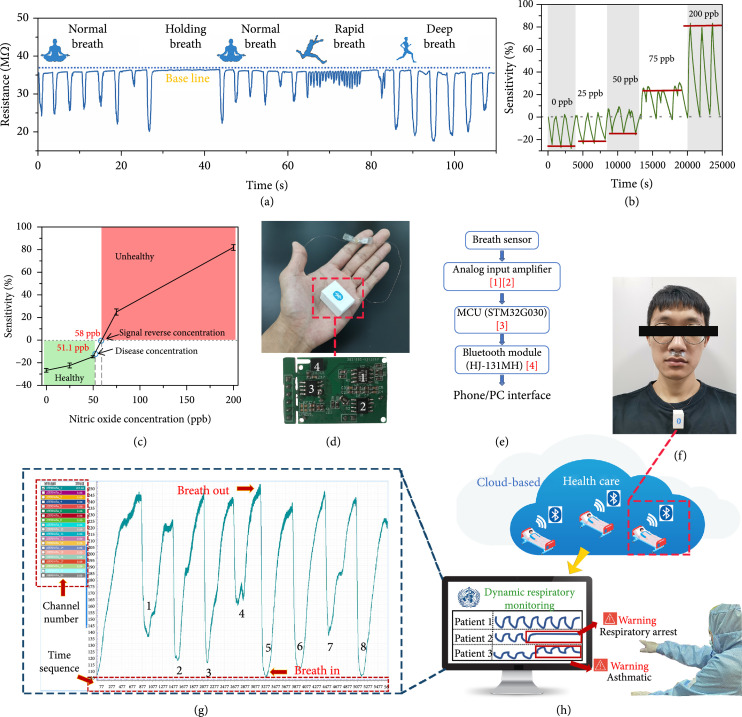
Possible breath monitoring and diagnoses using our epidermal wireless device in combination with the proposed cloud-based health care concept. (a) Monitoring of respiratory patterns (flow rate) using the epidermal LM@SnS_2_ sensor showing different breathing modes: normal breath, rapid breath, deep breath, and holding breath. (b) Simulating the sensing performance for different concentrations of NO. Note how the sensitivity reverses from negative to positive for NO concentrations ≥75 ppb. (c) Sensitivity as a function of different exhaled NO concentrations. (d) The epidermal wireless device and (e) the block diagram for data processing and delivery (the red numbers indicate the components enumerated in the inset of (d)). (f–h) Illustrating the concept of cloud-based multichannel telemonitoring of respiration; (f) placement on a volunteer; (g) example real-time breath monitoring showing the graphical user interface; (h) schematic illustrating the cloud-based remote diagnosis and monitoring approach.

To test the ability of our epidermal sensor to monitor the human respiratory activity, we recorded several breath patterns (Figure 5(a)). In healthy people, the resistance of the SnS_2_ on the electrode is reduced due to the humidity in the exhaled air. Clearly, the different periodicities and amplitudes in the electrical signal mirror the person’s respiratory patterns for different levels of activity, and we could distinguish between normal, rapid, and deep breathing and even identify when a person was holding their breath. These respiratory patterns cover nearly all breathing situations occurring in everyday life like resting, exercising, and even emergency situations like respiratory arrest. In addition, as out sensor simultaneously responds to the level of relative humidity and the concentration of NO gas, the device can be used for asthma patients. To simulate this, we simultaneously injected water vapor and NO gas into the test chamber, raising the relative humidity of the chamber from 60% (ambient level) to 85% (level in exhaled air) while varying the concentration of NO gas from 0 ppb to 200 ppb, to represent different severity levels of asthma. For NO concentrations <50 ppb, the sensitivity of the sensor is negative (Figure 5(b)) and the simulated waveform is consistent with the measured waveform from Figure 5(a). Once the NO concentration reached 75 ppb, the sensitivity changed from negative to positive, indicating the shift of SnS_2_ from an electron-doped to a hole-doped state. This change in sensitivity allows potential separation into healthy and unhealthy regions, which may be useful in a telediagnostic setting (Figure 5(c)). The reversal from negative to positive sensitivity occurs at an NO concentration of 58 ppb. In addition, the exhaled CO_2_ may also influence dope state of the SnS_2_. Since both CO_2_ and NO are oxidizing gases which would change the SnS_2_ from electron-doped to a hole-doped state. Thus, in practical uses, considering the effect of CO_2_, the reversal concentration of NO will be lower than 58 ppb which is closer to the clinical warning level. However, in the real breath monitoring process (Figure 5(a)), we did not observe the reverse of the signal with the presence of exhaled H_2_O and CO_2_, which further shows that the NO sensing function would not be affected by the presence of these two gases. In future clinical test with healthy people and typical patients, careful calibration should be carried out in order to eliminate the interference from these gases and fully satisfy the clinical use. The fractional exhaled nitric oxide (FeNO) concentration in healthy people typically ranges from 6.7 to 51.1 ppb and can increase to several hundred ppb in patients suffering from asthma [28, 63]. The upper limit of the healthy range (51.1 ppb) is close to the concentration at which the sensitivity changes sign (58 ppb). For practical applications, this means when the sensor is exposed to exhaled gas from people with a lung disease like asthma, the signal is reversed which allows the device to function as an early warning system for certain types of lung disease.

As a long-term and real-time monitoring device, it is important to minimize any adverse effects on a person’s normal daily activities. Wireless capabilities can expand the activity range of people subject to health monitoring using wearable devices. As a proof-of-concept, we developed a graphical user interface for the communication between our device and a laptop or phone, allowing data transmission in real-time for continuous breath monitoring and potential diagnostic and treatment decisions by a clinician (Figure 5(d)). This is illustrated with a block diagram in Figure 5(e) (for the detailed design, see Figure S17). The Microcontroller Unit (MCU) was 32-bit 8-pin packaged, used to design the prototype of the data processing system. The output voltage of the SnS_2_-based gas sensor was processed (analog filter) and amplified by an Op amp before being transmitted to a PC/phone via Bluetooth (HJ-131MH) at a rate of 460,800 Baud. The electronic device is powered by lithium batteries mounted on the back of the chip which greatly minimizes the size of the device (shown in Figure S18). The lithium battery is 1.5 V, and the model is CR1220.The real-time data is acquired via a custom-built application at the frequency of 250 Hz. The device weighs less than 11 g and is easy to wear. The epidermal sensing part can be directly attached to the philtrum while the processing unit can be worn around the neck (Figure 5(f)). The complete concept of the wireless communication and cloud-based processing is shown in Figures 5(g) and 5(h). We tested the device on a volunteer (Movie S1) and is showing an example output of eight complete breath cycles in Figure 5(g).

Hospitals with outpatients are one potential scenario for a possible application of the device. The multichannel monitoring interface is convenient for doctors to quickly obtain access to the respiratory status of all their patients. Asthma and respiratory arrest could trigger an automated alert. The patients’ respiratory rate and amplitude can be analyzed in real time from the dynamic waveform. The cloud-based design facilitates applications in modern medical Internet of Things (IoT) systems providing high degrees of accessibility, immediacy, and accuracy.

## 3. Conclusions

We have proposed a wireless wearable respiratory monitoring and diagnosis device with a design based on a liquid metal (LM) flexible electrode coupled to 2D SnS_2_ gas sensing nanomaterial. The deformability of LM provides the wearable sensor with excellent skin compatibility. The SnS_2_ sensing material exhibited a rapid response to changes in NO concentration capable of detecting changes at the ppb level. In addition, the material demonstrated a high selectivity and outstanding sensitivity, even when mechanically strained (being bent to simulate movement by the wearer) and in the presence of high levels of relative humidity (characteristic of exhaled human breath). We propose that the adsorption mechanism is governed by the combined effects of high adsorption energy, charge transfer, and changes in the two-dimensional SnS_2_ lamellar molecular structure. In addition, we integrated a Bluetooth terminal with the wearable sensor, which allows the real-time transmission to the wearer’s phone or PC and from there to the supervising clinician. The device is lightweight (less than 11 g) and has a negligible impact on daily activities. Through the cloud-based multichannel interface, the device can be used for real-time remote respiration monitoring and direct disease warning with great benefits to telemedicine applications allowing clinicians to simultaneously monitor several patients. The presented respiratory monitoring system holds much promise and can serve as a smart device for remote respiratory monitoring and diagnosis.

## 4. Materials and Methods

### 4.1. LM Electrode Fabrication

EGaIn (300 mg) and PVP (100 mg) were added into 10 ml ethanol; then, the mixture was sonicated by Ultrasonic Homogenizer (JY92-IIDN) with the power output setting to 50% (300 W) for 30 min. The temperature of the sample was controlled by using a cold-water bath in case of overheated. The gray liquid metal dispersion was then filtrated onto an organic filter membrane, followed by a mechanical sintering process using a metal roller. A laser of 355 nm (power: 35 W) was utilized to cut the membrane into the electrode shape. And the electrode was pasted to a PET film with thickness of 0.02 mm.

### 4.2. SnS_2_-Based Gas Sensor Fabrication

L-Cysteine (0.500 g and 4.0 mmol) and K_2_SnO_3_·3H_2_O (0.300 g and 1.0 mmol) were dissolved in 40 mL DI water. After stirring for 30 min, the mixture was transferred into a 50 mL Teflon-lined stainless steel autoclave and was heated to 200°C for 24 h. When cooling down to room temperature, the product was centrifuged with DI water for several times then vacuum dried at 60°C for 24 h. Finally, the yellowish brown powder was collected and dispersed in ethanol with the concentration of 20 mg/ml by ultrasonicator for 10 min. The sensing material was then drop-casted onto the LM electrode for further use.

### 4.3. Material Characterization

The morphology of the SnS_2_ was observed by scanning electron microscopy (SEM; HELIOS NanoLab 600i) and transmission electron microscopy (TEM; Tecnai G2 F30). The crystal structure was confirmed by X-ray diffractometer (XRD; Philips X’pert). The Raman spectrum was obtained by Via-Reflex under room temperature with excitation wavelength of 532 nm. UV-vis absorption spectrum was measured using PE Lambda 950. The morphology of LM nanoparticle was observed by SEM (Phenom Scientific).

### 4.4. Gas Sensing and Optoelectronic Performance

The resistance of the sensor was recorded by a multimeter. The homemade chamber for simulating the real breath environment was made with a transparent acrylic board, and the volume was 8 L (20cm×20cm×20cm). The air was acted as the background gas, and the concentration of target gas was controlled by injection certain volume of high concentration gas into the chamber by a high precision syringe. A ceramic heater (3cm×3cm) was placed in the chamber, and the RH was controlled by evaporating a certain amount of water by the heater. For the investigation of the optoelectronic and light sensitivity performance, the lasers of different wavelengths and power densities, which were bought from Yunxiang Co., Ltd., were vertically irradiated through transparent acrylic plate right onto the sensing material. The tests of the epidermal electrodes and wearable sensors on human skin were approved by all participants and Ethics Board of Shenzhen Institutes of Advanced Technology (approval number: SIAT-IRB-170320-YGS-HJP-A0340).

### 4.5. Theoretical Calculations

All the calculation was performed by the package DMol3 in the Material Studio software. The Local Density Approximate (LDA) with Perdew-Wang (PWC) function was chosen for structural relaxations and total energy calculation. A 4×4×1 supercell was built, and 2×2×1Γ centered Monkhorst–Pack grid for the Brillouin zone sampling was operated, which gave the converged results of all properties. A 15 Å vacuum region was added, which was large enough to weaken the effects of the periodic images. The convergence threshold of the maximum energy changes was $1.0\times 10^{-5}$ Ha/per atom, the maximum force was 0.002 Ha/Å, and the maximum displacement was 0.005 Å.

## Data Availability

The data used to support the findings of this study are available from the corresponding author upon request.

## Supplementary Materials

Supplementary 1 Figure S1: size distribution of liquid metal nanoparticle in ethanol. Figure S2: the resistance changes of the liquid metal wire with the deformation of skin. Figure S3: the resistance changes of the liquid metal wire in 15 days. Figure S4: the band structure diagram. Figure S5: the response of SnS_2_ sensor to water vapor under room temperature. Figure S6: the dynamic response to 21% concentration of O_2_. Figure S7: the dynamic response to CO_2_. Figure S8: the adsorption model of CO molecule onto SnS_2_ layer. Figure S9: the adsorption energy and charge transfer of CO molecule onto SnS_2_ layer. Figure S10: the temperature effect on the resistance of the sensor. Figure S11: plot of (Ah*ν*)_2_ against (h*ν*) of SnS_2_ by UV-vis spectra. Figure S12: time-resolved photo response under blue (450 nm), green (532 nm), and red (650 nm) light of 1 mW/cm^2^. Figure S13: temporal photocurrent response under blue light of various power densities (1, 3, 5, 10, 20, and 40 mW/cm^2^). Figure S14: fitting curve of photocurrent and power density under blue light illumination. Figure S15: amplification factor of SnS_2_ under different wavelengths and power densities of light illumination. Figure S16: the closest adsorption distance between target gas molecules and SnS_2_ supercell layer. Figure S17: block diagram of detailed circuit design. Figure S18: the wireless chip powered by lithium batteries. Scheme S1: the homemade gas sensing testing and photo detection testing platform. Table S1: comparison between reported NO gas sensors and present work.

Supplementary 2 Movie S1: wireless real-time breath monitoring.
