# Supplementary material for: Liquid Metal-Based Epidermal Flexible Sensor for Wireless Breath Monitoring and Diagnosis Enabled by Highly Sensitive SnS2 Nanosheets
Source: Research (Wash D C). 2021 Jun 17;2021:9847285. doi: 10.34133/2021/9847285 (PMC11014675; doi:10.34133/2021/9847285)
Supplement: Supplementary 1 — Figure S1: size distribution of liquid metal nanoparticle in ethanol. Figure S2: the resistance changes of the liquid metal wire with the deformation of skin. Figure S3: the resistance changes of the liquid metal wire in 15 days. Figure S4: the band structure diagram. Figure S5: the response of SnS2 sensor to water vapor under room temperature. Figure S6: the dynamic response to 21% concentration of O2. Figure S7: the dynamic response to CO2. Figure S8: the adsorption model of CO molecule onto SnS2 layer. Figure S9: the adsorption energy and charge transfer of CO molecule onto SnS2 layer. Figure S10: the temperature effect on the resistance of the sensor. Figure S11: plot of (Ahν)2 against (hν) of SnS2 by UV-vis spectra. Figure S12: time-resolved photo response under blue (450 nm), green (532 nm), and red (650 nm) light of 1 mW/cm2. Figure S13: temporal photocurrent response under blue light of various power densities (1, 3, 5, 10, 20, and 40 mW/cm2). Figure S14: fitting curve of photocurrent and power density under blue light illumination. Figure S15: amplification factor of SnS2 under different wavelengths and power densities of light illumination. Figure S16: the closest adsorption distance between target gas molecules and SnS2 supercell layer. Figure S17: block diagram of detailed circuit design. Figure S18: the wireless chip powered by lithium batteries. Scheme S1: the homemade gas sensing testing and photo detection testing platform. Table S1: comparison between reported NO gas sensors and present work. [file 9847285.f1.docx]

Supplementary Materials

Liquid Metal based Epidermal Flexible Sensor for Wireless Breath Monitoring and Diagnose Enabled by Highly Sensitive SnS_2_ Nanosheets

Yifan Huang, Fan Yang, Sanhu Liu, Rongguo Wang*, Jinhong Guo*, Xing Ma*





***Figure S1. Size distribution of liquid metal nanoparticle in ethanol.***

***Scheme S1. The home-made gas sensing testing and photo detection testing platform.***


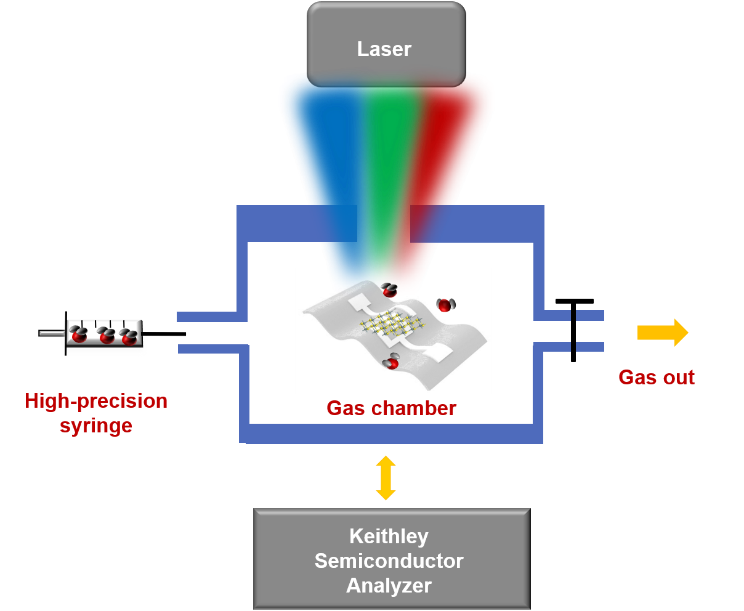


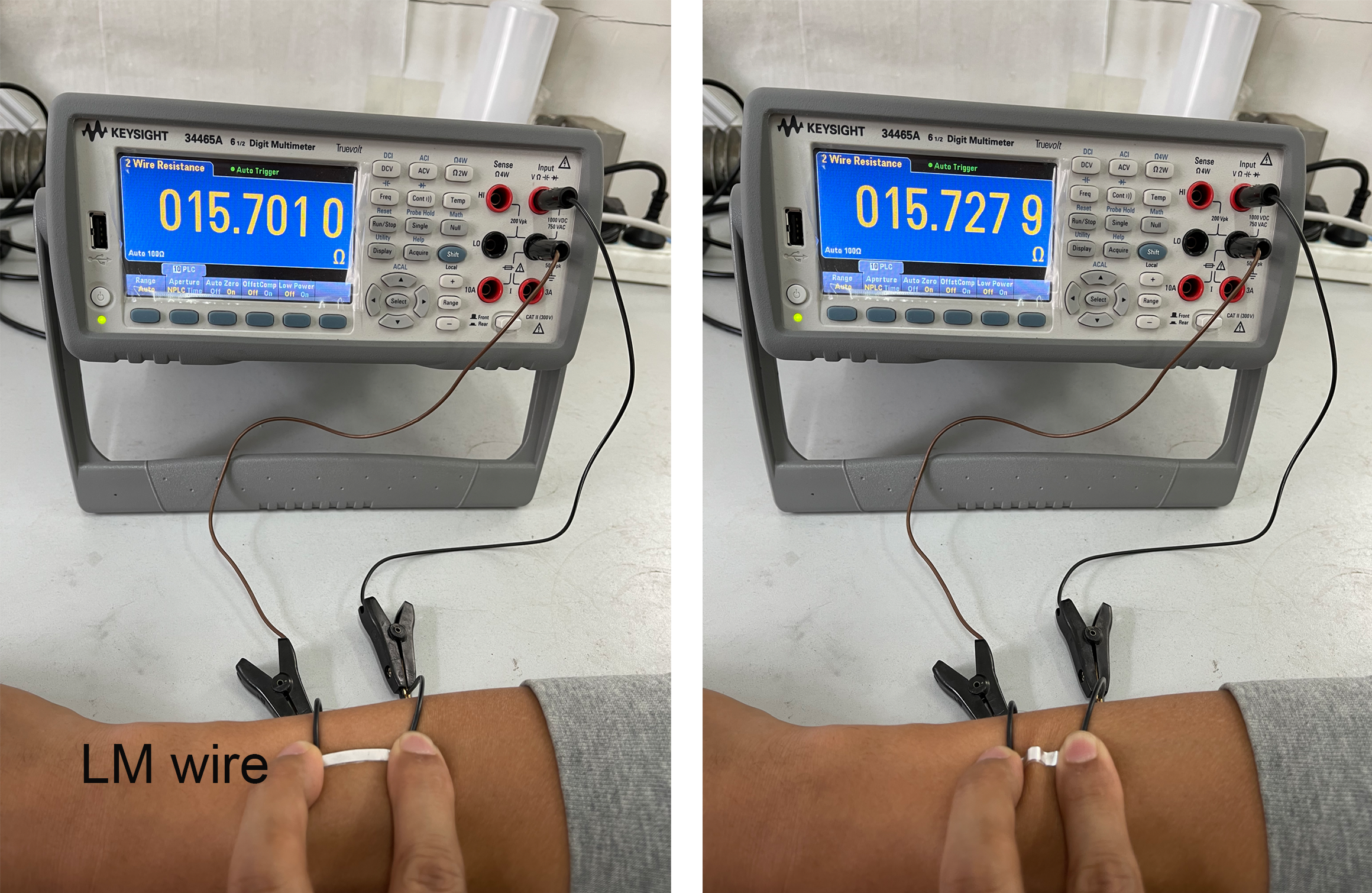


***Figure S2 The resistance change of the liquid metal wire with the deformation of skin.***





***Figure S3 The resistance changes of the liquid metal wire in 15 days***

Figure S2 provides the band structure of EGIA and SnS_2_. The work function of SnS_2_ is reported to be 5.1eV. The EGIA is composed by 75% Ga and 25% In, and the work function of Ga and In are 4.20eV and 4.12eV, respectively. The work function of EGIA is reported to be 4.1-4.2eV (as shown in Figure S4a). Since the work function of EGIA is higher than that of SnS_2_, ohmic contact is forming (as shown in Figure S2b). When the target gas is injected, NO can further gain electrons from SnS_2_ to form NO^-^; thus, the Femi level is moving toward the conduction band. This is also true to H_2_O gas, which tend to lose eletrons to form H_2_O^+^; thus, the Femi level is moving toward the valence band.


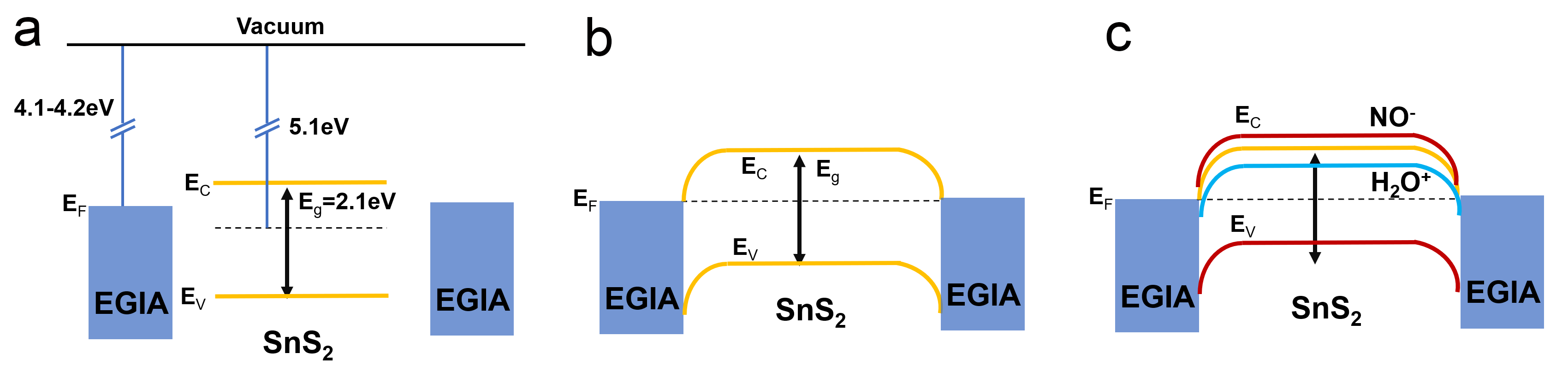


***Figure S4. The band structure diagram*** *a) before EGIA electrode and SnS_2_ contact; b) after EGIA electrode and SnS_2_ contact; c) when the SnS_2_ react with targe gases.*





***Figure S5. The response of SnS_2_ sensor to water vapor under room temperature.*** *The environment relative humidity (RH) is 60%. The RH in the testing chamber was controlled by evaporating a certain amount of DI water to increase the RH. And recovery process was operated by open the testing chamber, and the RH of the chamber recovery to environment RH.*

The concentration of O_2_ and CO_2_ in air are about 21% and 0.03%, respectively. While the concentration of O_2_ and CO_2_ in exhaled gas are about 16% and 4%, respectively. In order to explore the influence of O_2_ concentration changes on the performance of the sensor, we put the sensor in an airtight box and continuously fill it with N_2_, in which way the oxygen concentration of the environment where the sensor is located is expected to be reduced from 21% to about 0%. The dynamic sensing signal is shown in Figure S4. The response to 21% concentration changes of O_2_ is about 7.5%. And the response to O_2_ in exhaled gas (only about 5% concentration changes) should be lower than 7.5%, also much lower than the response to 25ppb of NO. Therefore, the change of oxygen concentration has little effect on the sensing performance.


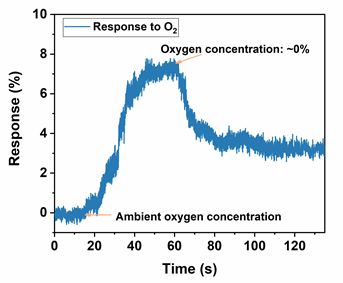


***Figure S6 The dynamic response to 21% concentration of O_2_.***

To investigate the influence of CO_2_ concentration changes, we tested the response to 4% concentration of CO_2_ (the concentration changes from ambient environment to exhaled gas). As shown in Figure S7a, it shows the same type of response as NO. That is, with the injection of CO_2_, the resistance of the sensor increased. The response is calculated to be about 62% (shown in Figure S7b). In our device, the NO warning function is realized based on the different redox types of water vapor and NO. When we only consider the effects of water vapor and NO on the sensing response, as shown in Figure 5c, the resistance signal of the sensor will reverse from decreasing trend to increasing trend when the NO concentration improves to around 58 ppb. As reported, the exhaled NO concentration of healthy people is 5.0 -51.1 ppb, and the median concentration is around 20 ppb. Since CO_2_ and NO are both oxidizing gases, when we consider the effect of three gases together: CO_2_, H_2_O and NO, the reversing concentration of NO will decrease due to the contribution from the other two gases and therefore becoming closer to the medium concentration of NO (20 ppb). So, the warning function of the device should be more accurate in practical uses. Besides, in the real breath monitoring process (Figure 5a), we did not observe the reverse of the signal with the presence of exhaled H2O and CO_2_, which further shows that the NO sensing function would not be affected by the presence of the two gases. In future clinical test with healthy people and typical patients, careful calibration should be carried out in order to eliminate the interference from these gases and fully satisfy the clinical use.


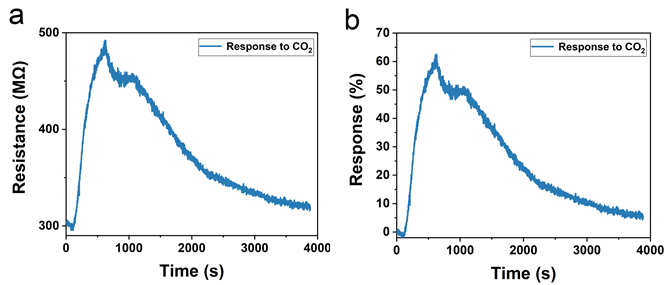


***Figure S7*** *(a)* *the dynamic resistance change; (b) the dynamic response of the sensor to 4% concentration of CO_2_.*


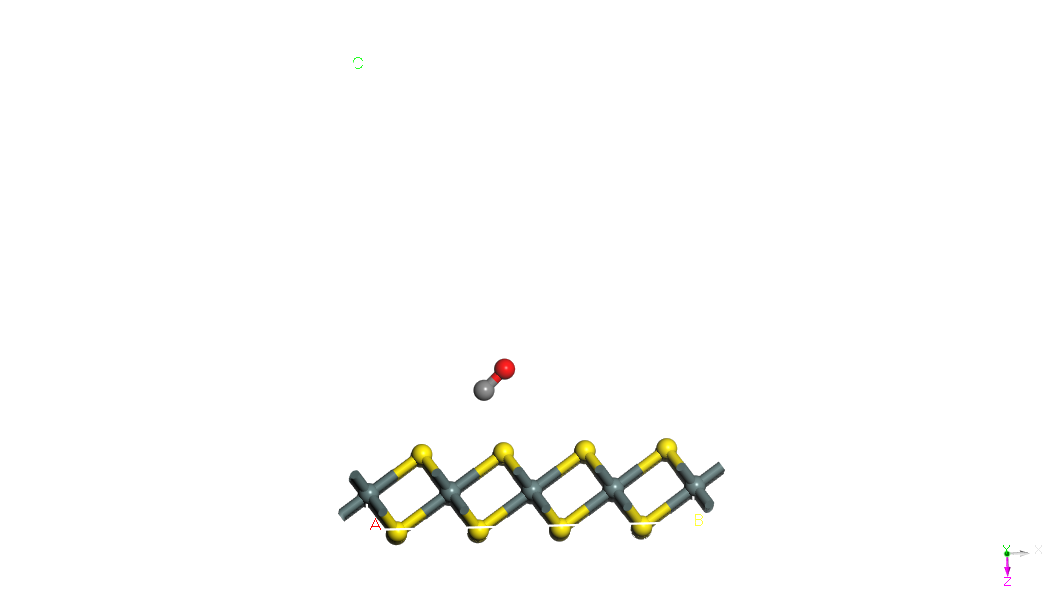


***Figure S8 The adsorption model of CO molecule onto SnS_2_ layer***


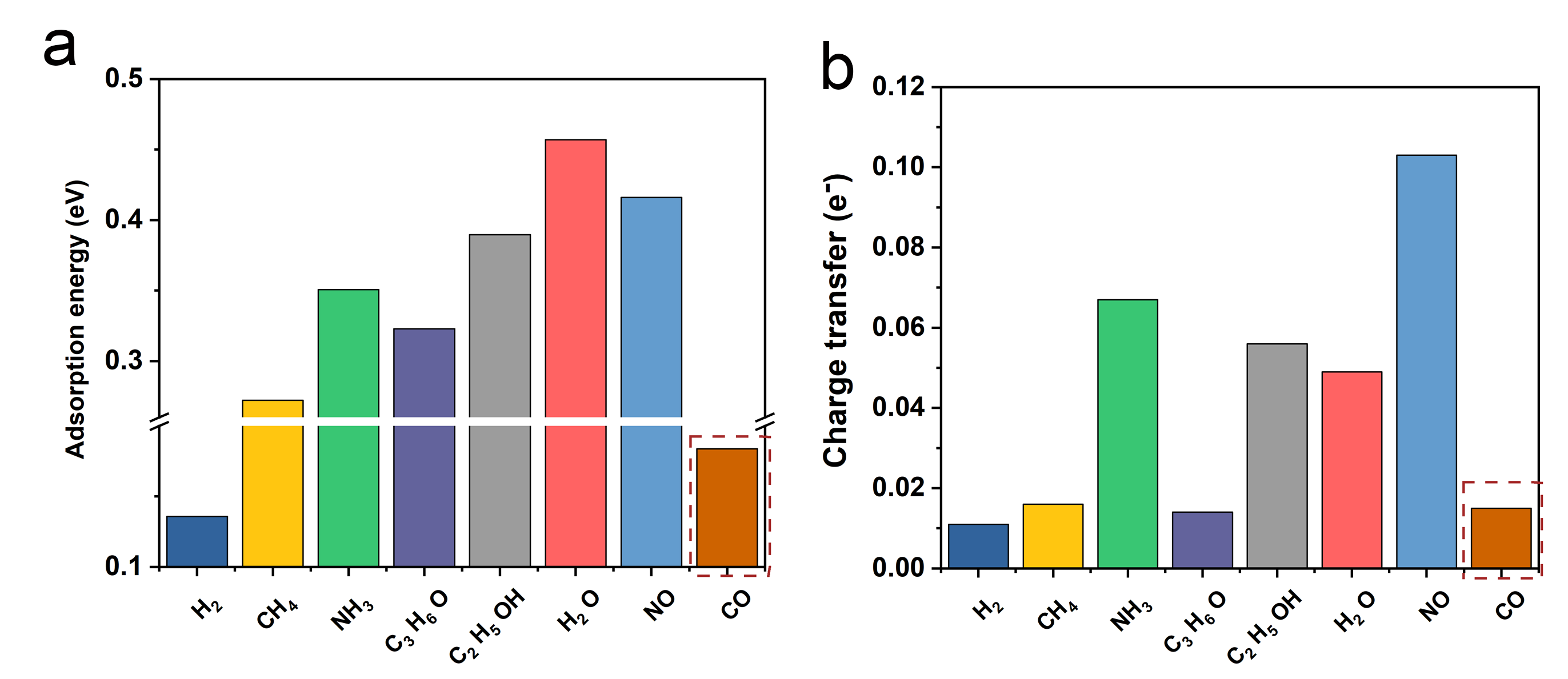


***Figure S9 (a) The adsorption energy and (b) charge transfer of CO molecule onto SnS_2_ layer***


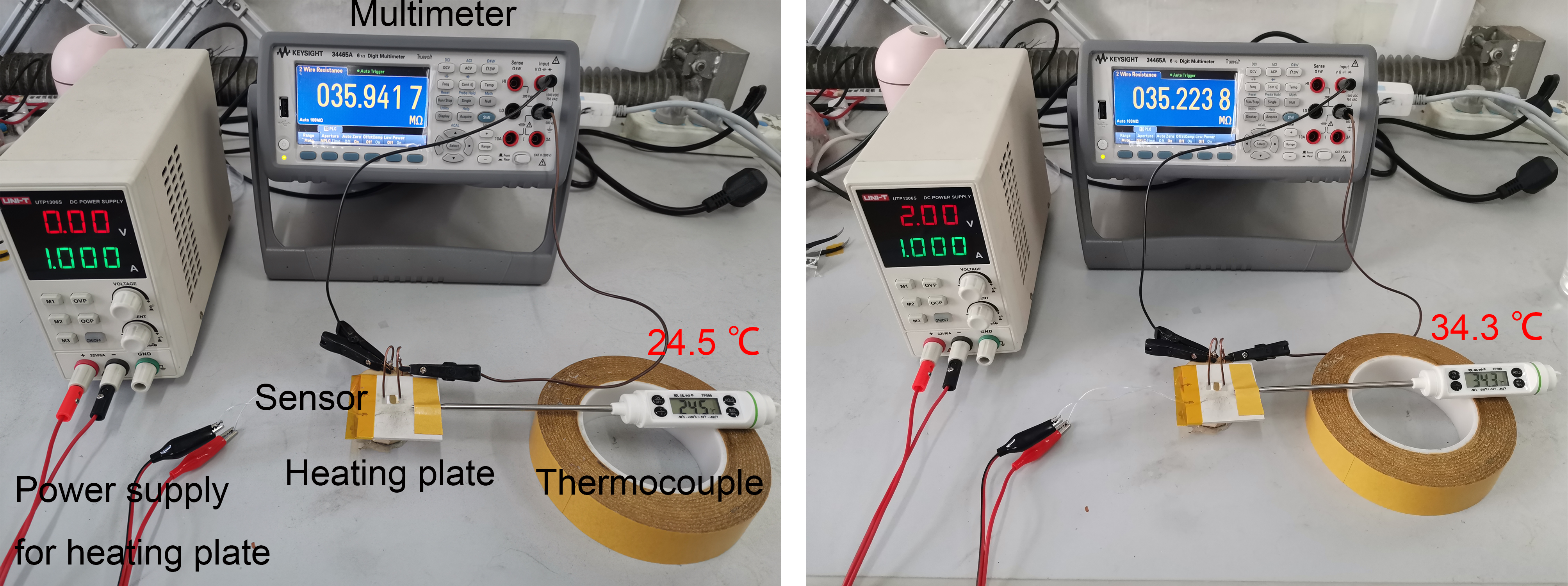


***Figure S10 The temperature effect on the resistance of the sensor.***


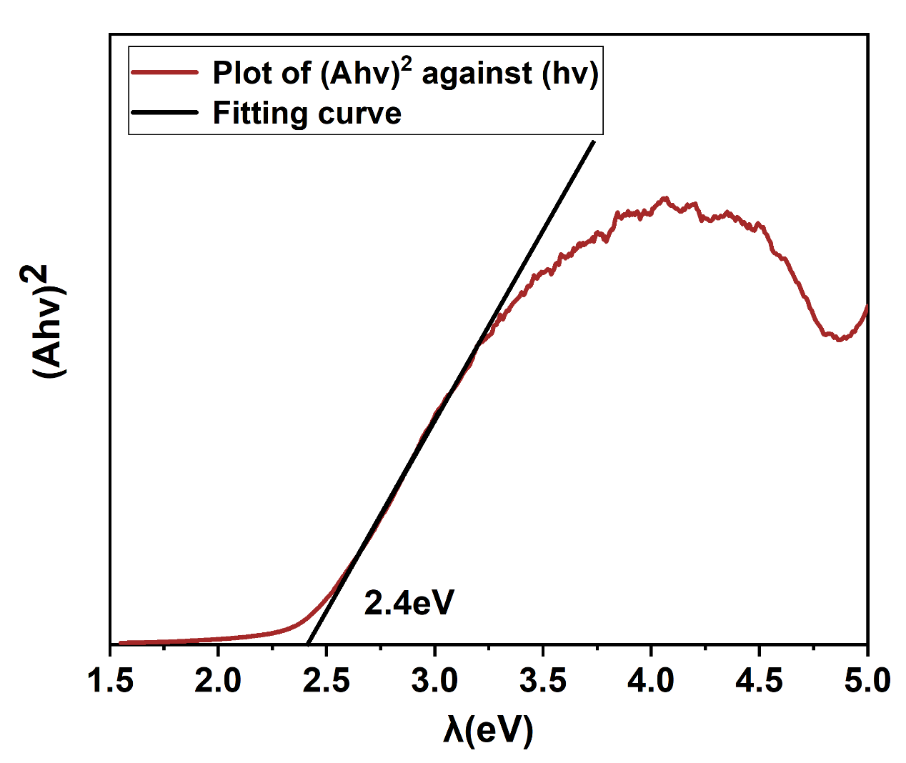


***Figure S11. Plot of (Ahν)^2^ against (hν) of SnS_2_ by UV−vis spectra.*** *The band gap of the SnS_2_ layer was confirmed to be 2.4 eV.*

Transient photodetection testing was operated by chopping the relevant light with 1 mW/cm^2^ repeatedly, and the on- and off-current did not show clear-cut distinctions in five cycles, indicating good stability and reproducibility. In addition, this device performed a significant response to wide range of wavelength and blue light triggers the largest response. More photogenerated carriers would be produced by blue light, owing its higher photon energy among the three chosen light, causing larger photocurrent based on the function: E = 1.24/λ, where E is the energy of single photon, and λ represents the wavelength of the light.





***Figure S12. Time-resolved photo response under blue (450 nm), green (532 nm), and red (650 nm) light of 1 mW/cm^2^.***


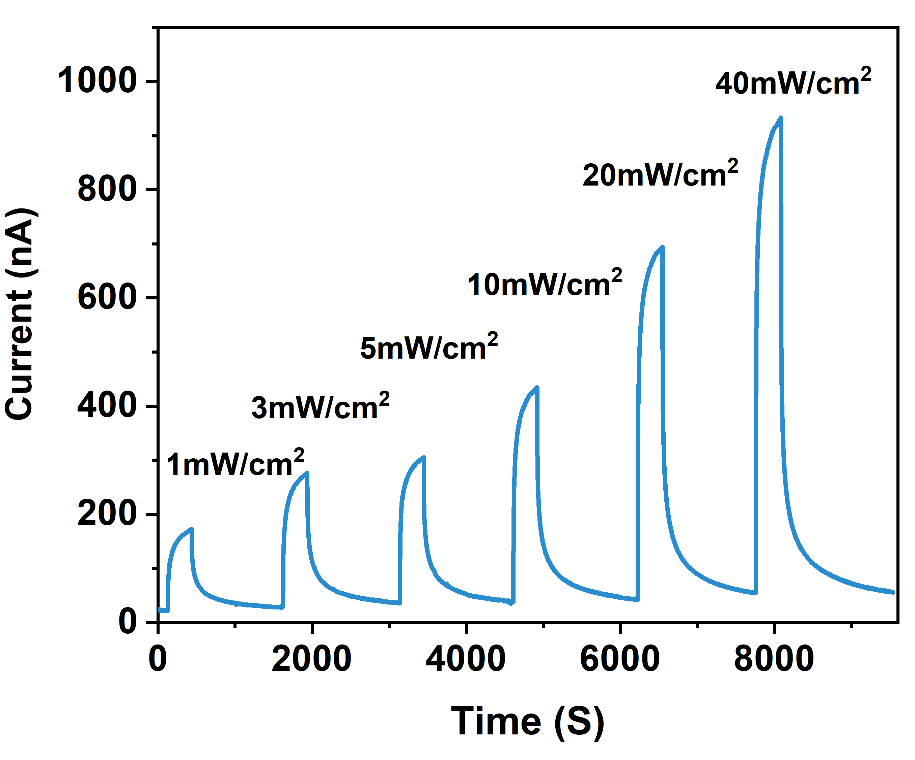


***Figure S13. Temporal photocurrent response under blue light of various power density (1, 3, 5, 10, 20 and 40 mW/cm^2^).***

We ascribe the relationship between photocurrent and power density by: Ip = AP^θ^, where Ip, A, P, θ represent the photocurrent, constant, light power density, and empirical value, respectively. The equation is calculated to be Ip = 143.55 × P^0.51^, performing excellent sublinear fitting with R^2^ = 0.9906.


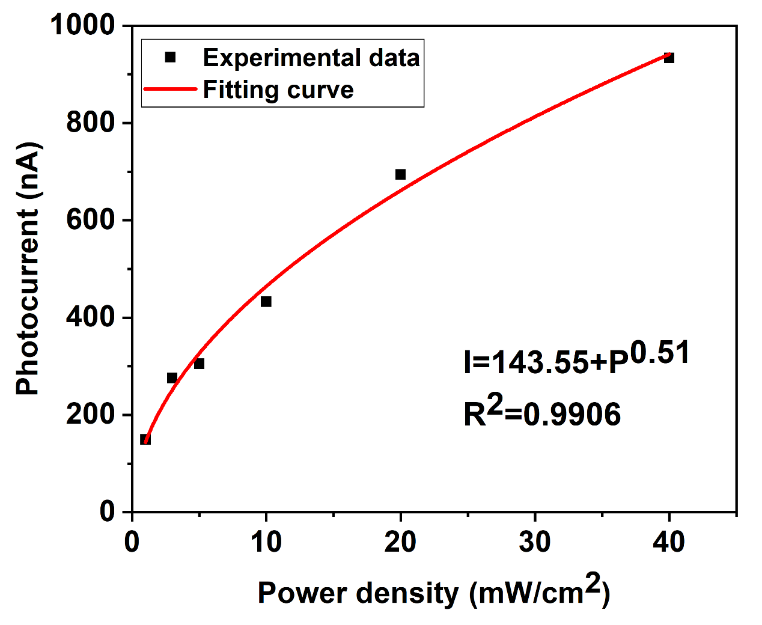


***Figure S14. Fitting curve of photocurrent and power density under blue light illumination.***



Considering the photogenerated carriers transferring to the conduction band and easily combining with target gas, we exposed the device to the three lasers, and adjusted the power density (1, 3, 5 mW/cm^2^) to investigate the light illumination effect on gas-sensing behavior. We defined the amplification factor (AF) as S_light_/S_dark_, where S_light_ and S_dark_ represent the sensitivity under light-on and dark, respectively. Clearly, AF higher than 1 indicates the enhancement effect, while lower than 1 means the opposite. Surprisingly, both blue and green light which produced the much higher photocurrent than red light, show a similar trend of slightly enhancement to rapid weakening with the increase of light power density. And the sensitivity under green light of 5 mW/cm^2^ dropped to nearly one-fifth of that under dark. However, the red light causing the weakest photocurrent boosted the sensitivity most and the optimum peak located at 3mW/cm^2^ where the device exhibited as high as 2.14 folds magnified sensitivity. The phenomenon is attributed to the pre-adsorption of oxygen in the air and the physical desorption of the oxygen anion induced by light irradiation. Also, the sensitivity enhancement performance relies on the equilibrium motivation of adsorption and desorption process rather than the stronger photoinduced carriers.

***Figure S15. Amplification factor of SnS_2_ QD/rGO nanohybrids under different wavelengths and power densities of light illumination.*** *The sensor was exposed to 200ppb NO gas under 60% RH and room temperature. Red light with 3mW/cm^2^ improved the sensitivity most by 2.14 folds. Green light with 5 mW/cm^2^ weakened the sensitivity most, droping to 0.22 of the sensitivity under dark.*





***Figure S16. The closest adsorption distance between target gas molecules and SnS_2_ supercell layer.*** *NO and H_2_O molecules exhibited the shortest distance among the interference gas.*


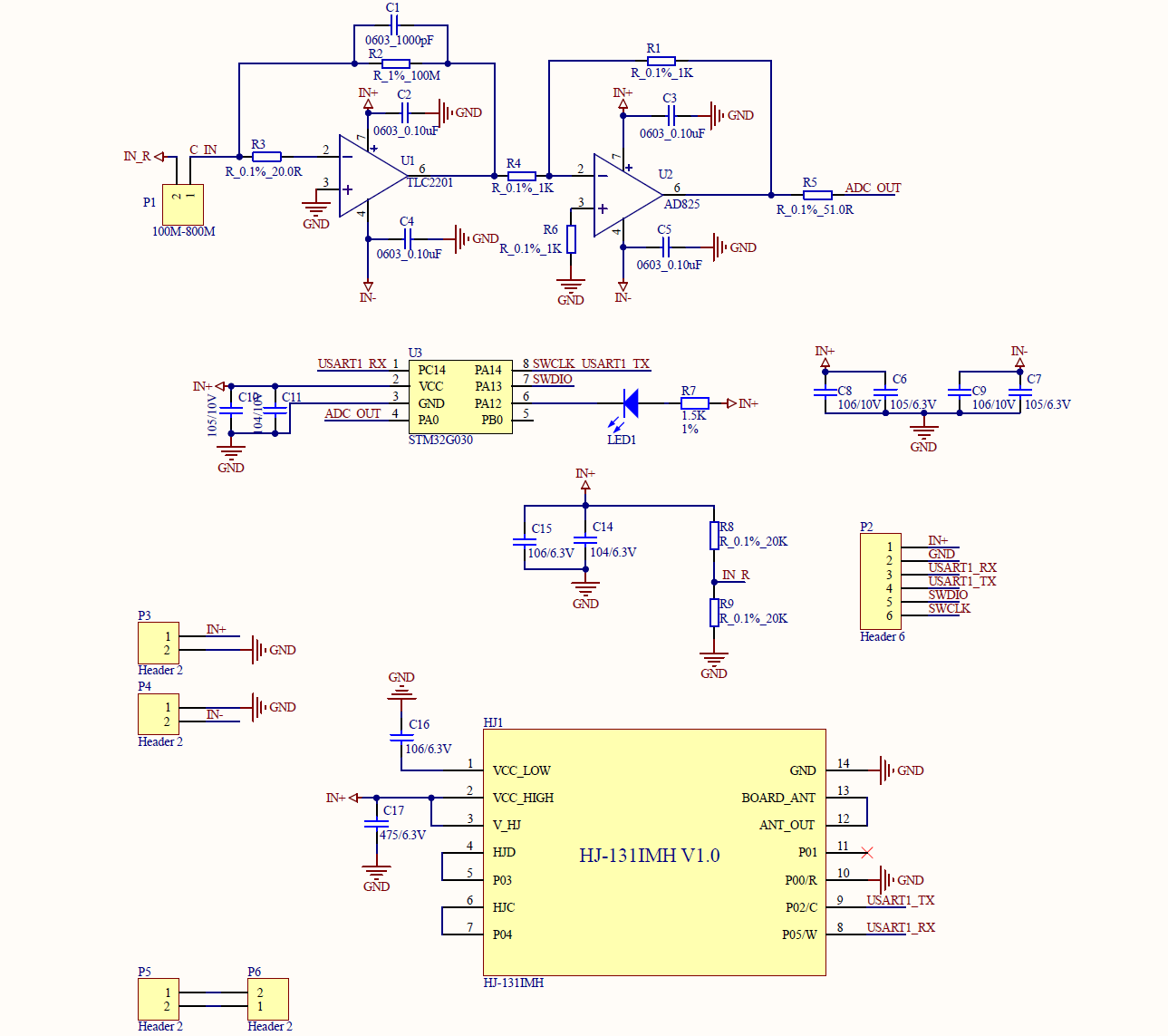


***Figure S17. Block diagram of detailed circuit design.***


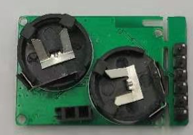

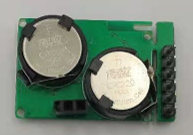


***Figure S18. The wireless chip powered by lithium batteries.***

***Table S1*** *Comparation between reported NO gas sensors and present work*

| Material | Operation temperature  [℃] | Sensitivity  [%/ppm] | Experimental LOD  [ppb] | Reference |
| --- | --- | --- | --- | --- |
| ZnO/CdO | 215 | 38 | 3000 | [43] |
| SnO_2_ | 200 | 1611.8 | 100 | [47] |
| WSe_2_ | 25 | 313 | 72 | [46] |
| MoS_2_ | 25 | 36.7 | 800 | [41] |
| Ag/ZnO | 200 | 900 | 7.2 | [42] |
| SnS_2_ | 25 | 2336 | 15 | This work |
